# Threatened Caribbean Coral Is Able to Mitigate the Adverse Effects of Ocean Acidification on Calcification by Increasing Feeding Rate

**DOI:** 10.1371/journal.pone.0123394

**Published:** 2015-04-15

**Authors:** Erica K. Towle, Ian C. Enochs, Chris Langdon

**Affiliations:** 1 Rosenstiel School of Marine and Atmospheric Science, University of Miami, Miami, Florida, 33149, United States of America; 2 Atlantic Oceanographic and Meteorological Laboratories (AOML), National Oceanographic and Atmospheric Administration (NOAA), Miami, Florida, 33149, United States of America; GEOMAR Helmholtz Centre for Ocean Research Kiel, GERMANY

## Abstract

Global climate change threatens coral growth and reef ecosystem health via ocean warming and ocean acidification (OA). Whereas the negative impacts of these stressors are increasingly well-documented, studies identifying pathways to resilience are still poorly understood. Heterotrophy has been shown to help corals experiencing decreases in growth due to either thermal or OA stress; however, the mechanism by which it mitigates these decreases remains unclear. This study tested the ability of coral heterotrophy to mitigate reductions in growth due to climate change stress in the critically endangered Caribbean coral *Acropora cervicornis* via changes in feeding rate and lipid content. Corals were either fed or unfed and exposed to elevated temperature (30°C), enriched pCO_2_ (800 ppm), or both (30°C/800 ppm) as compared to a control (26°C/390 ppm) for 8 weeks. Feeding rate and lipid content both increased in corals experiencing OA vs. present-day conditions, and were significantly correlated. Fed corals were able to maintain ambient growth rates at both elevated temperature and elevated CO_2_, while unfed corals experienced significant decreases in growth with respect to fed conspecifics. Our results show for the first time that a threatened coral species can buffer OA-reduced calcification by increasing feeding rates and lipid content.

## Introduction

Rising atmospheric carbon dioxide from anthropogenic sources is driving the oceans toward conditions not seen for millions of years and will ultimately have strong negative repercussions for corals on a global scale [1]. This elevated atmospheric CO_2_ has increased global average temperature by 0.2°C decade^-1^ over the last 30 years, with much of that heat being absorbed by the ocean [2]. Increased warming throughout this century may reach a pointwhere excessive coral bleaching, the expulsion of corals’ symbiotic dinoflagellates, may cause widespread mortality. In addition to absorbing heat, the ocean has absorbed one-third of the CO_2_ produced by anthropogenic activities. This has resulted in a decrease of 0.02 pH units decade^-1^ over the last 30 years, a phenomenon known as ocean acidification (OA) [1]. This decrease in pH is accompanied by a decline in the saturation state (Ω) of calcium carbonate which impairs the ability of corals and other calcifying organisms to form skeletons [3]. Corals will need to expend more energy to achieve a constant rate of calcification as the saturation state of the ambient seawater decreases due to OA [47,48]. Less is known about how combined warming and OA will affect the coral holobiont, but recent work has shown that temperature can modulate the response of coral physiology to OA—in some cases mitigating OA effects, and in some cases worsening OA effects [36,49], highlighting the need for improved understanding of these interactions. Growth and survivorship of corals in the future will depend on their resilience capability in the face of climate change stress.

Identifying potential indicators of resilience to climate change stress has been a major focus of coral physiologists during the last decade. Anthony et al. [4] showed that a coral’s capacity to utilize heterotrophy (feeding) and lipid content were good predictors of survivorship during a bleaching event in the Pacific coral *Acropora intermedia* because lipids stored in coral tissue represent significant energy reserves that can be used in times of stress [5, 6]. Lipids in corals can be translocated from their symbionts or obtained directly from feeding [7]. Heterotrophy may be a major source of lipids for corals because some essential fatty acids cannot be synthesized *de novo* [6], and heterotrophy may become more significant when a coral bleaches and loses its primary source of daily metabolic energy from its symbionts [8]. Anthony et al. [9] predicted that coral survival following a bleaching event would be strongly influenced by remaining lipid reserves and rates of heterotrophy.

Increasing the energy available to corals by enhanced heterotrophy and lipid reserves may mitigate the negative impacts of climate change stressors like OA [10, 11] and warming [8]. Thermal bleaching results in reduced symbiont density and chlorophyll *a* levels [12], and recent studies have shown that OA stress may also decrease symbiont density [13,14]. Very little is currently known about how lipids may affect a coral’s response to OA; however, it logically follows that corals that can increase their feeding rates and lipid reserves may be better able to cope with and recover from stressors that may reduce the amount of photosynthate they receive from their symbionts. Certain species may be more capable of heterotrophy than others [8,15], but there is a paucity of information regarding the ability of particular Atlantic coral species to utilize heterotrophy as a means to ameliorate the deleterious effects of warming and OA, as much of the seminal work on this topic has been done on Pacific corals. This lack of data is especially problematic for the critically threatened Staghorn coral (*Acropora cervicornis*), which was once one of the dominant reef-building coral species in the western Atlantic and Caribbean [16]. Since the late 1970’s, unprecedented declines in *A*. *cervicornis* populations [17] have been documented in virtually all areas of the western Atlantic and Caribbean [18]. In the Florida Reef Tract, populations have been diminished by as much as 98% [19]. In order to successfully inform conservation decisions, it is imperative to understand physiological responses to stress and potential for resilience of the species chosen for restoration efforts.

The primary objectives of this study were to test if heterotrophy can mitigate reductions in growth due to climate change stress (both warming and OA), and to determine if feeding rate and total lipid content are plastic responses that change significantly under stress in *A*. *cervicornis*. The effect of heterotrophy and lipids on corals is documented for a small number of species; however, most studies generally had a “fed” and “unfed” group, but did not directly quantify ingestion rates. We identify a stress buffering mechanism, i.e. the ability to plastically increase ingestion rate and total lipid content that provides the coral with extra energy. This extra energy may be used to offset reductions in growth that would otherwise result from reduced photosynthate transfer, in the case of thermal stress, or increased energy demands on calcification, in the case of OA stress.

## Materials and Methods

### Collection and experimental design

Due to the conservation status of *A*. *cervicornis*, collection of wild colonies is not permitted. Therefore, eight colonies of *A*. *cervicornis* were donated from three South Florida sources in May 2013: the Smithsonian Institute (Ft. Pierce, FL), the University of Miami Coral Resource Facility, and an *A*. *cervicornis* nursery near Broad Key, FL. The purpose of using colonies from three sources was to maximize genetic diversity across populations and individuals in this study. All colonies were taken from approximately 5m depth. Corals were fragmented into three-five cm experimental units and affixed to aluminum gutter guard using All Game epoxy. Replicates were haphazardly distributed to avoid a parental colony effect and allowed to recover from fragmentation under control conditions (26°C/390 ppm (LT-LCO_2_)) for four weeks prior to the beginning of the study. This study consisted of four treatments: 26°C/390 ppm (LT-LCO_2_), 26°C/800 ppm (LT-HCO_2_), 30°C/390 ppm (HT-LCO_2_), and 30°C/800 ppm (HT-HCO_2_) and lasted eight weeks from June to August 2013. Each treatment was replicated twice for a total of eight independent tanks, and ten corals (five fed and five unfed throughout the experiment) were in each tank. While N = 2 tanks for each experimental condition may appear low, there is precedent for this design [11]. We believe that our design represents a reasonable tradeoff between the competing needs of replication and the space and cost limitations of adding additional experiment units. The HT level was chosen because it is immediately below mean bleaching threshold in the Florida Keys (30.4°C, [20], and the HCO_2_ level was chosen as that predicted for the year 2065 (800 ppm, [21], RCP 8.5). Corals were held in semi-recirculating tanks throughout the duration of the experiment. Carbonate chemistry was manipulated by direct gas injection and was monitored via direct measurement of total alkalinity (TA) and dissolved inorganic carbon (DIC).

### Aquaria set-up

Experimental corals were maintained at the Climate Change Laboratory at the University of Miami in 45 l tanks of water replenished by a 250 l sump tank with complete water turnover every ten minutes. The high quality natural seawater supply for the tanks came from intakes in nearby Bear Cut, Key Biscayne, FL. This water was filtered to ten microns, therefore the corals in this study were not receiving significant nutrition from plankton introduced to their tanks by the seawater supply. Each sump tank contained a heating and cooling element connected to a temperature controller (OMEGA CN7533) with accuracy within 0.1°C. CO_2_ levels were achieved by bubbling the sump tanks with CO_2_-enriched air produced using mass flow controllers (Sierra Instruments model 810C). Corals experienced natural light attenuated by a neutral density shade cloth to produce light levels similar to those experienced at donor locations, but still within the range of a typical Florida patch reef environments. Daily integrated PAR averaged 5.8 mol photons m^-2^ d^-1^. The average peak midday instantaneous PAR was 353±70 μmol photons m^2^ s^-1^. This light level is consistent with previous work on coral physiology and heterotrophy done at ~300 μmol photons m^-2^ s^-1^ [44,49].

### Seawater chemistry

In order to monitor seawater chemistry conditions throughout the study, discrete water samples were taken from each tank weekly and poisoned with mercuric chloride to be analyzed for total alkalinity (TA) and dissolved inorganic carbon (DIC). TA was measured in duplicate on an automated Gran titrator and standardized using certified reference materials obtained from Dr. A. Dickson (Scripps IO). DIC was measured in duplicate using a DIC analyzer (Apollo SciTech Inc.) and standardized to the same certified reference seawater. Mean temperature, salinity, TA, and DIC were used to calculate pCO_2_, pH, and aragonite saturation state (Ω_a_) for each treatment using the program CO_2_SYS using K_1_ and K_2_ from Mehrbach et al. [22] refit by Dickson and Millero [23] per Lewis and Wallace [24].

### Heterotrophy

In order to understand the role of heterotrophy in ameliorating thermal and OA stress effects on growth rate and lipid content, corals in the study were divided equally into a fed and unfed group. Fed corals were placed in a plastic container and fed a diet of dried zooplankton powder (Ziegler’s Larval AP 100) *ad libitum* twice a week. Unfed corals were placed in a similar container without food for the same period of time so as to receive similar handling without the nutrition. The objective was to obtain a group of corals that had to get all their nutrition from their zooxanthellae and any plankton that might get through the 10 micron water filtration system, and another group that uniformly received a supplemental source of nutrition if they were able to feed heterotrophically.

### Feeding rate

In order to learn if *A*. *cervicornis* changes its feeding behavior under conditions of thermal and OA stress, we conducted feeding rate assays where the capture rate of live rotifer prey was measured on just the fed corals in each of the treatment tanks. These measurements were made four times (biweekly) on each fed coral at two, four, six and eight weeks into the experiment. Feeding rate assays were performed on days when the corals did not receive their normal twice weekly feeding with powdered zooplankton. The amount of live rotifer prey captured likely contributed an insignificant amount to the lipids of the fed corals. The purpose of the feeding rate assay was to provide information about the feeding intensity of the corals and how that feeding behavior varied with treatment. Each coral was placed in 1 l, well-stirred beaker of treatment water containing 10,000 live rotifers. After 1 hour the coral was removed and the number of rotifers remaining in the beaker was enumerated. The prey capture or feeding rate was expressed as the number of rotifers removed per cm^2^ of coral surface area per hour. The rotifer species, *Brachionus plicatilis*, was chosen for this study. This species is widely used as nutritious live food for the raising of larval fish, invertebrates and coral. A rotifer concentration of 10,000 l^-1^ was selected for our experiments. This concentration is approximately 5-times the natural zooplankton concentration reported for local reef waters of 1,700 zooplankters l^-1^ (Leichter et al. [28]). Sebens et al. [29] has recommended the use of prey concentrations up to 10-times natural prey densities in feeding rate studies so as to avoid a concentration dependence that can confound the interpretation of results. The two part feeding protocol was meant to maximize the chance of seeing treatment effects that may be small. The twice weekly *ad libitum* feeding with powdered zooplankton maximized our power to observe a heterotrophic feeding impact on long-term measures of coral condition, i.e. growth and total lipid content. The short-term live rotifer prey capture assays allowed us to probe for treatment effects on the heterotrophic feeding behavior of the corals. Doing these assays at a prey density that is likely saturating maximized our chances of seeing a treatment effect that could be masked if the capture rates were confounded by a prey concentration effect.

The details of the live prey feeding rate assays were as follows. Initial and final concentrations of live rotifers were measured and feeding rates were calculated following Coughlan [25]. Eleven one l beakers were used, ten containing a coral and one control without coral to account for any possible changes in rotifer density not due to coral feeding. Each beaker had the same flow rate (controlled by magnetic stir bar), light conditions, and initial rotifer density from the same stock solution. Corals were allowed to feed for one hour after sunset as in Grottoli et al. [8] and were observed to have extended tentacles in the presence of rotifers, indicating feeding was occurring. Initial concentrations of rotifers were approximately 10,000 cells l^1^. After one hour, four replicate fifteen ml water samples were taken from each beaker, fixed in the preservative Lugols solution, and final rotifer concentration was quantified via microscopy. Experiments took place on days when corals were not fed the dried zooplankton diet. An advantage of this method over the gut excavation method published by Palardy et al. [26, 27] and Grottoli et al. [8] is that it is non- destructive allowing us to make feeding rate, growth, chlorophyll *a*, total lipid and symbiont density measurements on all the corals in the study, rather than just a subset.

### Coral growth and tissue lipid content

Growth rates were measured biweekly as changes in coral weight in air using the buoyant weight technique according to Davies et al. [30]. At the end of the study, coral tissue was removed from the skeleton using an air-brush and homogenized following Szmant et al. [31]. The aliquot for total lipids (2 mL) was filtered onto a GF/A filter and frozen at -80°C until further analysis following Teece et al. [6]. Briefly, a 2 ml aliquot of total coral homogenate was extracted three times (4 ml 1:1 dichloromethane:methanol). The resulting organic extracts were collected, after vortexing; all three extracts were combined, dried under a stream of nitrogen gas, and weighed on an analytical balance. 3D scanning methodologies for surface area calculations followed Enochs et al. [32].

### Symbiont density and chlorophyll *a* concentration

One ml of total blastate was filtered onto a GF/A filter for chlorophyll *a* analysis and frozen at -80°C until further analysis following Holm-Hansen and Riemann [33]. Chlorophyll *a* samples were analyzed on a fluorometer (TD-700 Turner Designs) calibrated with purified Chl *a* (Sigma-Aldrich catalog no. C6144). Pigment content was normalized to coral surface area. Another one ml subsample of the total blastate was preserved with 50 μl of Lugols solution for endosymbiont quantification, which was calculated using two independent replicate counts on a haemocytometer (Hausser Scientific) using VistaVision compound microscope at 100× magnification. Symbiont density was normalized to surface area.

### Statistics

All statistical analyses were completed in the program JMP version 11.0.0. Normality and homoscedasticity were ascertained prior to testing each dependent variable using a Shapiro-Wilk test and Levene’s test, respectively. For the dependent variables: growth, lipids, chlorophyll *a*, and symbiont density, a three-way full factorial mixed ANOVA model was run with temperature (26° vs. 30°), CO_2_ (390 ppm vs. 900 ppm)_,_ and nutrition (fed vs. unfed) as fixed factors, and replicate tanks nested within temperature and CO_2_ level to account for any tank effect. For growth and lipids, there was no significant tank effect (α = 0.05), and thus data from replicate tanks were pooled. For chlorophyll *a* and symbiont density, there were significant tank effects, thus replicate tanks were not pooled for these two dependent variables. For the dependent variable feeding rate, only fed corals were assessed, thus a two-way full-factorial mixed ANOVA model was run with temperature and CO_2_ as fixed factors and tank nested within temperature and CO_2_ level to account for any tank effects. For feeding rate, there were no significant tank effects (α = 0.05), so data from replicate tanks were pooled. Where effects were found to be significant, a *post-hoc* Tukey’s HSD test was run to determine which means were different.

### Ethics Statement

Eight parent colonies of *Acropora cervicornis* (Invertebrate Cnidarian) were donated from aquaria or coral nurseries in the State of Florida, USA, in May 2013, and this study was fully approved and funded by MOTE Marine Laboratories “Protect Our Reefs” Grant (#POR-2012-22) and the Mohamed bin Zayed Species Conservation Fund (Project #12054710). As a scientific organization, University of Miami’s Rosenstiel School of Marine and Atmospheric Science’s Corals and Climate Change laboratory is empowered to conduct studies of this nature.

## Results

**Fig 1:**
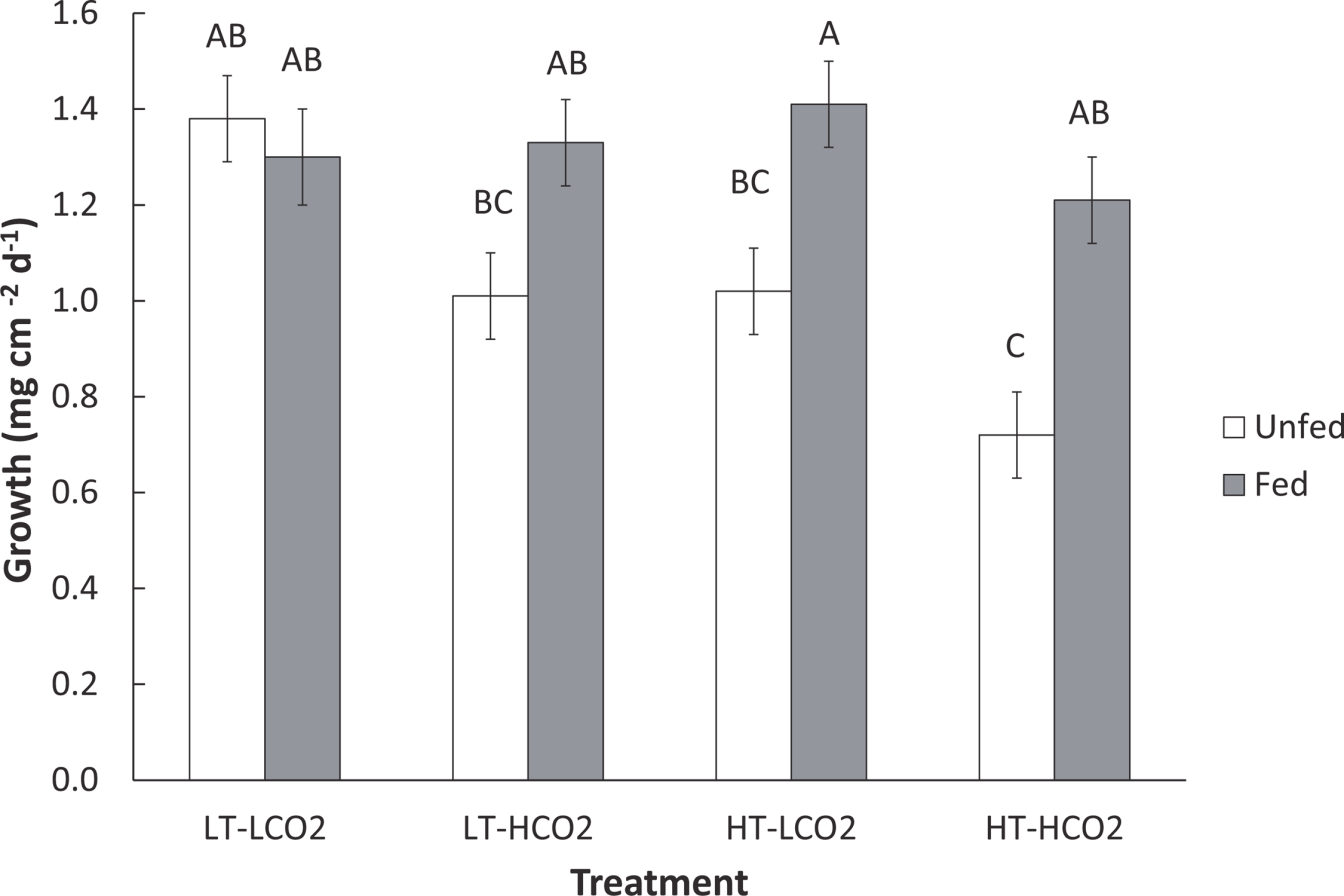
Growth of *A*. *cervicornis* over 8 week exposure to combinations of temperature, CO_2_, and feeding. LT-LCO_2_ represents control conditions, 26°C, 390ppm, LT-HCO_2_ represents 26°C, 800ppm, HT-LCO_2_ represents 30°C, 390 ppm, and HT-HCO_2_ represents 30°C, 800ppm. Each bar represents the mean growth rate of n = 10 corals, and white bars represent unfed corals, while grey bars represent fed corals. Dissimilar letters indicate means that are significantly different following a *post-hoc* Tukey’s HSD test. Error bars represent ± one standard error.

**Fig 2:**
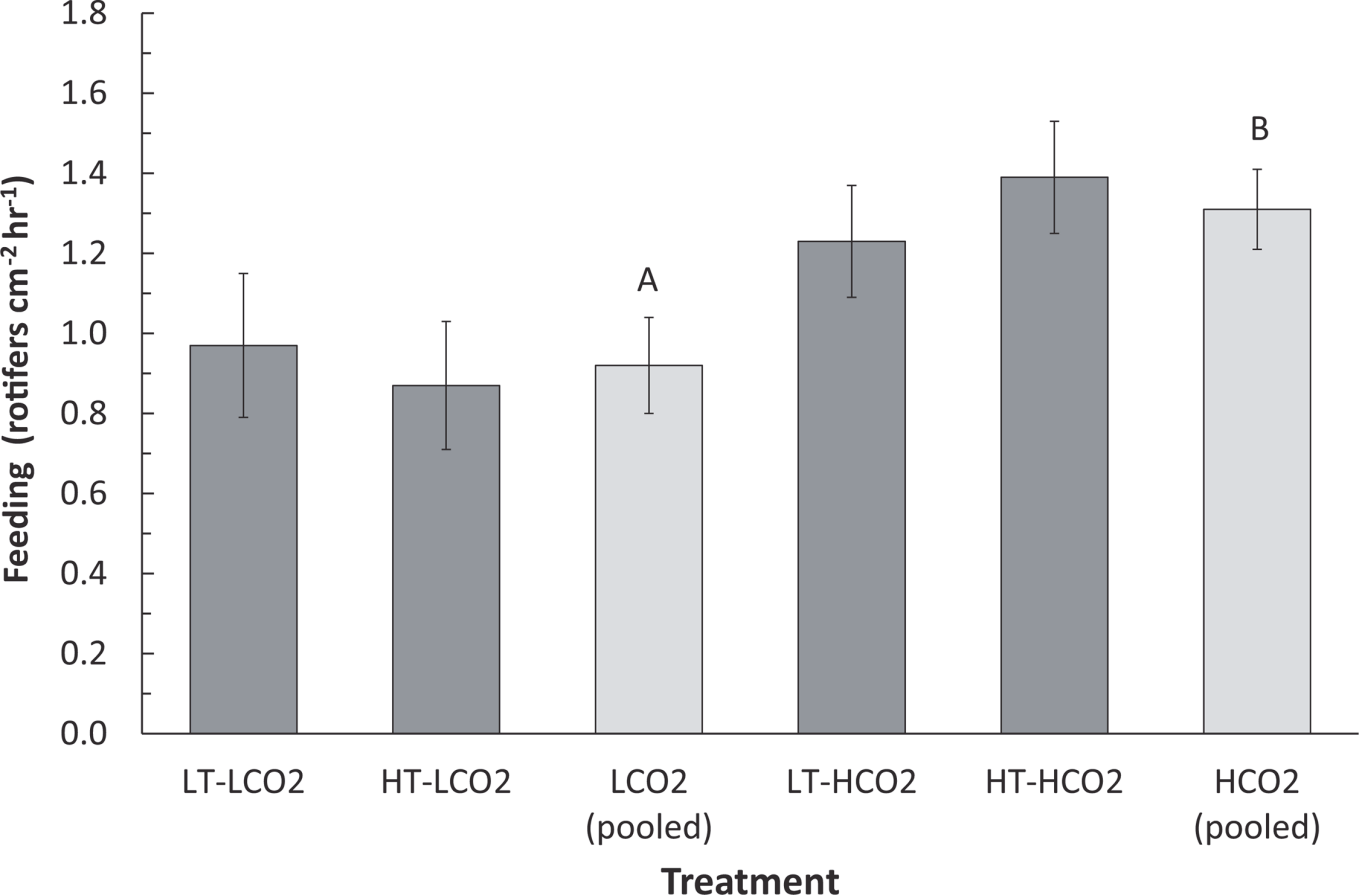
Feeding rate of *A*. *cervicornis* over 8 week exposure to combinations of temperature and CO_2_. LT-LCO_2_ represents control conditions, 26°C, 390ppm, HT-LCO_2_ represents 30°C, 390 ppm, LT-HCO_2_ represents 26°C, 800ppm, and HT-HCO_2_ represents 30°C, 800ppm. Each dark grey treatment bar represents the mean feeding rate of n = 10 corals, while light grey LCO_2_ and HCO_2_ bars (pooled by temperatures) representing n = 20 corals are shown to clearly depict the main effect of CO_2_ on feeding rate. Dissimilar letters indicate means that are significantly different following *post-hoc* student’s t-test. Error bars represent ± one standard error.

**Fig 3:**
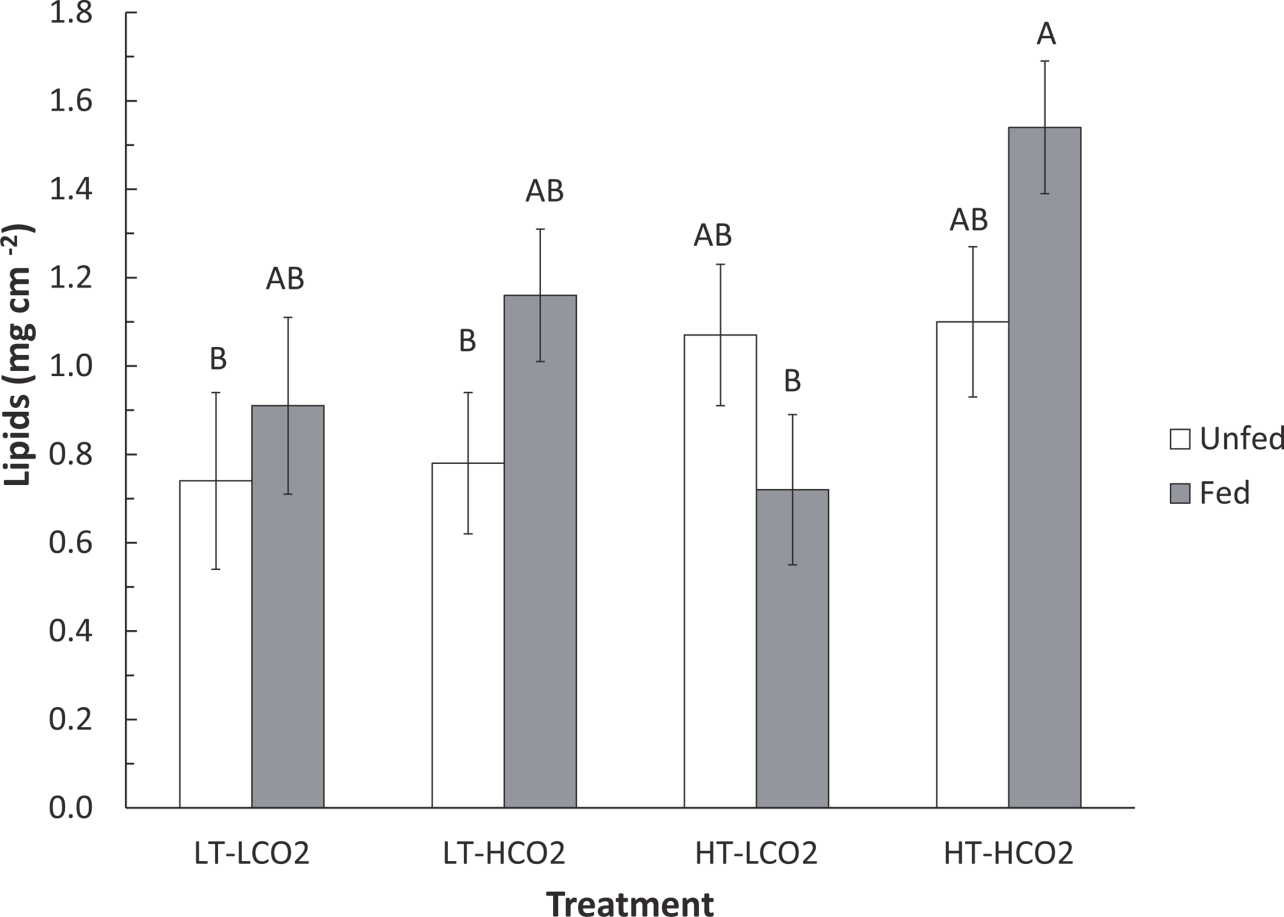
Total lipid content of *A*. *cervicornis* following 8 week exposure to combinations of temperature, CO_2_, and feeding. LT-LCO_2_ represents control conditions, 26°C, 390ppm, LT-HCO_2_ represents 26°C, 800ppm, HT-LCO_2_ represents 30°C, 390 ppm, and HT-HCO_2_ represents 30°C, 800ppm. Each bar represents the mean lipid content of n = 10 corals, and white bars represent unfed corals, while grey bars represent fed corals. Dissimilar letters indicate means that are significantly different following a *post-hoc* Tukey’s HSD test. Error bars represent ± one standard error.

**Fig 4:**
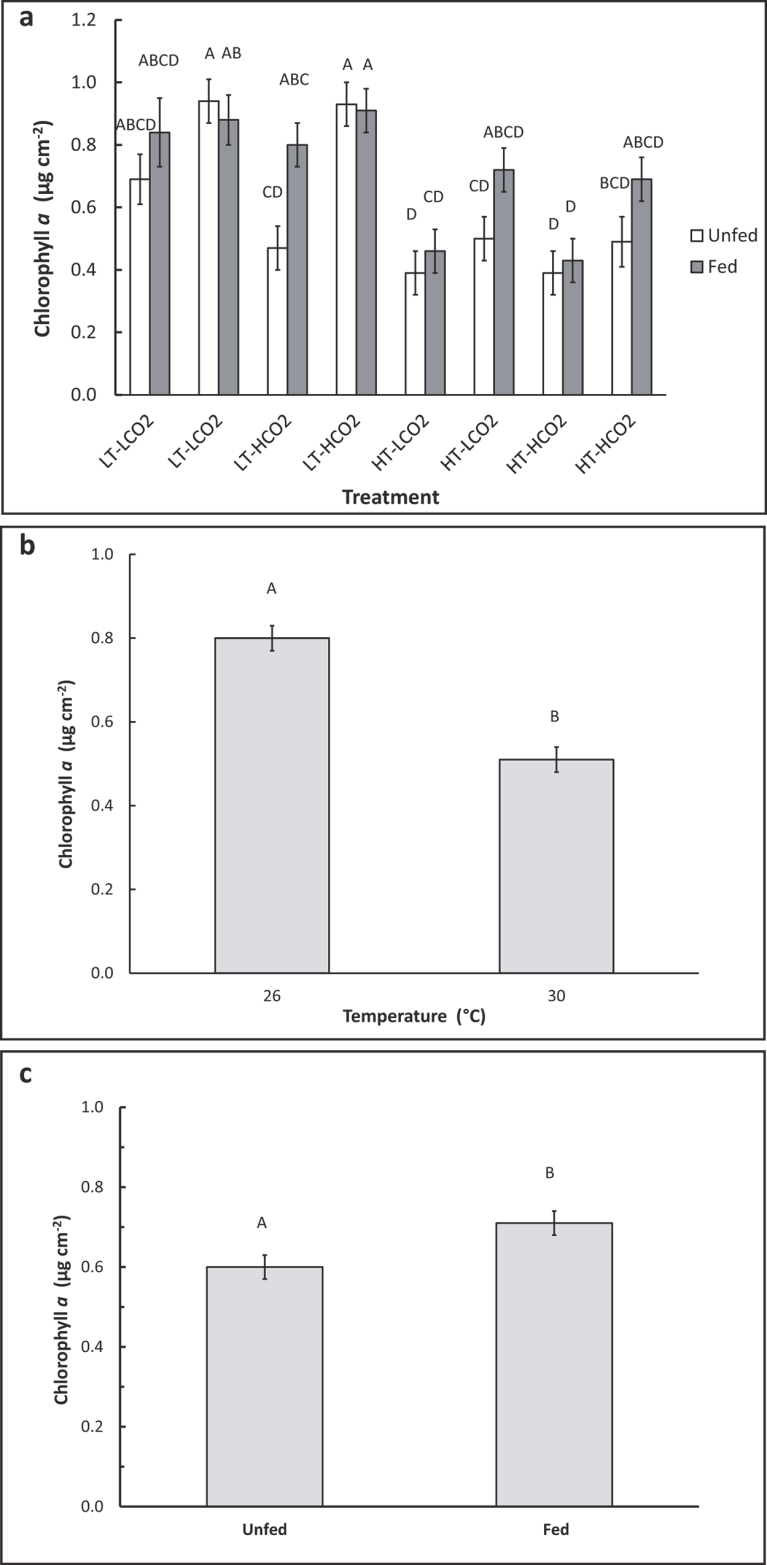
Chlorophyll *a* level in *A*. *cervicornis* following 8 week exposure to combinations of temperature, CO_2_, and feeding. A. Mean chlorophyll *a* of corals (n = 10) in each individual tank, not pooled by treatment due to a significant tank effect. Therefore each treatment is shown twice, representing each replicate tank, i.e. two LT-LCO_2_, two LT-HCO_2_, two HT-LCO_2_, and two HT-HCO_2_, from left to right. Dissimilar letters indicate means that are significantly different following *post-hoc* Tukey’s HSD test. Mean chlorophyll *a* of corals (each bar represents n = 40) depicting the main effects of temperature (B) and feeding (C). Dissimilar letters indicate means that are significantly different following *post-hoc* student’s t-test. Error bars represent ± one standard error.

**Fig 5:**
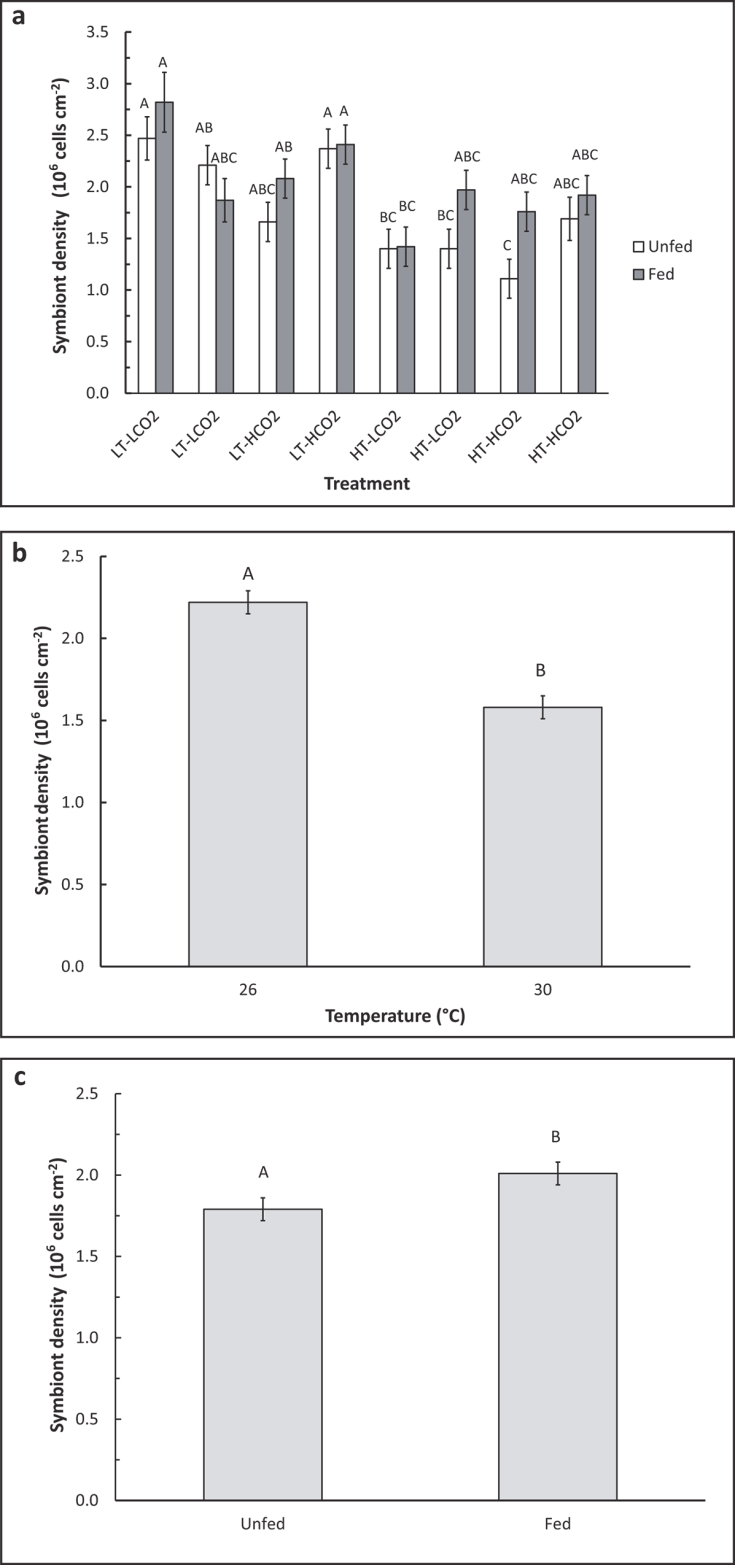
Symbiont density in *A*. *cervicornis* following 8 week exposure to combinations of temperature, CO_2_, and feeding. Mean symbiont density of corals (n = 10) in each individual tank, not pooled by treatment due to a significant tank effect. Therefore each treatment is shown twice, representing each replicate tank, i.e. two LT-LCO_2_, two LT-HCO_2_, two HT-LCO_2_, and two HT-HCO_2_, from left to right. Dissimilar letters indicate means that are significantly different following *post-hoc* Tukey’s HSD test. Mean chlorophyll *a* of corals (each bar represents n = 40) depicting the main effects of temperature (B) and feeding (C). Dissimilar letters indicate means that are significantly different following *post-hoc* student’s t-test. Error bars represent ± one standard error.

**Fig 6:**
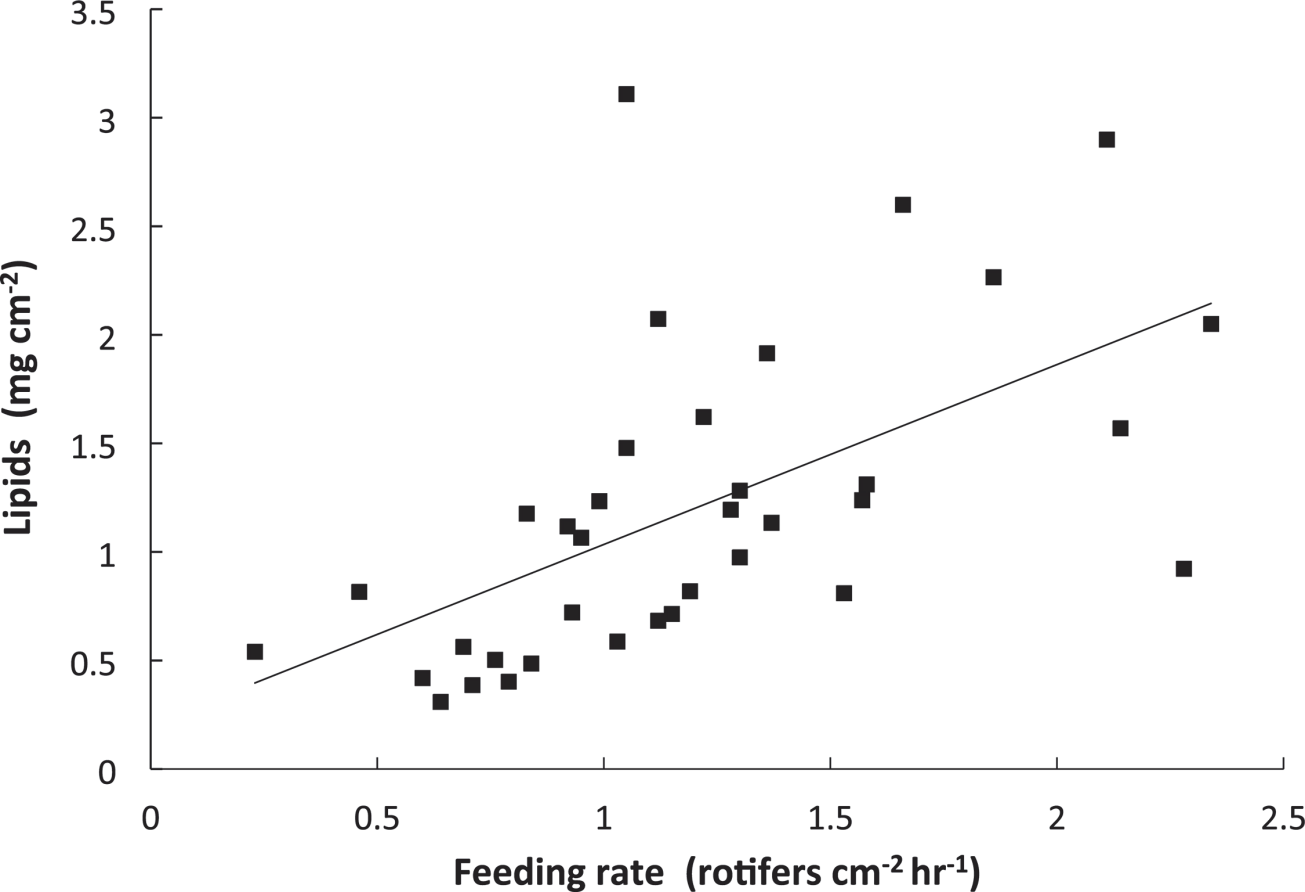
Bivariate linear fit of lipid content by feeding rate in *A*. *cervicornis*. Linear fit (p = 0.0002) for n = 36 fed coral feeding rates and their corresponding lipid contents. The equation of the best fit line is y = 0.82869(x) + 0.2058, with a R^2^ value of 0.3355.

Temperature, CO_2_, and other water chemistry parameters are presented in Table 1. A three–way full-factorial ANOVA revealed that temperature, CO_2_ and feeding each had significant main effects on growth rate (Table 2), e.g. HT decreased growth, HCO_2_ decreased growth, and feeding increased growth. There was a significant interaction between feeding and temperature on growth (Table 2, F(1,78) = 6.64, p = 0.012), which is reflected in similar growth rates of fed corals under LT and HT conditions, but reduced growth rates of unfed corals under HT conditions when compared to LT conditions (Fig 1). Growth of unfed HT-HCO_2_ was significantly lower than the controls, and growth of unfed corals was also significantly lower than fed conspecifics in HT treatments at both CO_2_ levels (Fig 1). Feeding rate was significantly affected by CO_2_ (Table 2, F(1,34) = 4.44, p = 0.045). Feeding rate was approximately 30% higher in HCO_2_ treatments (1.31 rotifers hr^-1^ cm^-2^) than in LCO_2_ treatments (0.92 rotifers cm^-2^ hr^-1^), regardless of temperature (Fig 2). Temperature did not have a significant effect on feeding rate. Total lipid content was affected by CO_2_ (Table 2, F(1,66) = 5.63, p = 0.021) as well as by the interaction between CO_2_ and feeding (Table 2, F(1,66) = 4.14, p = 0.047). Fed corals in the HT-HCO_2_ treatment had significantly greater lipids than unfed controls (Fig 3). Temperature had no significant effect on lipid content. Chlorophyll *a* and symbiont density were significantly affected by replicate tank (Table 2, F(1,74) = 7.49, p <.0001) (Table 2, F(1,74) = 4.52, p = 0.003) respectively, and therefore replicate tanks were not pooled by treatment. Means of all eight tanks are displayed in Fig 4 for chlorophyll *a* and Fig 5 for symbiont density, and the significant main temperature effect (Table 2, F(1,74) = 55.11, p<.0001) (Table 2, F(1,74) = 40.21, p<.0001), respectively, can be visualized. Corals at 30°C had less chlorophyll *a* content than corals at 26°C (Fig 4B). Feeding also had a significant effect on chlorophyll *a* (Table 2, F(1,74) = 8.18, p = .006) whereby unfed corals had less chlorophyll *a* than fed corals (Fig 4C). Corals at 30°C had lower symbiont density than corals at 26°C (Fig 5B). Feeding also had a significant effect on symbiont density (Table 2, F(1,74) = 5.05, p = 0.028) whereby unfed corals had lower densities than fed corals (Fig 5C). CO_2_ did not have a statistically significant effect on chlorophyll *a* levels or symbiont density in *A*. *cervicornis* in this study. The reason for the significant tank effect on chlorophyll *a* and symbiont density is unknown, but this effect was not large enough to preclude seeing significant main effects of temperature and feeding on these parameters, nor was it large enough to be significant for growth, feeding rate, or lipid content measurements. A bivariate linear fit was tested for lipid content and feeding rate, and was found to be significant, (Table 3, R = 0.34, p = 0.0002) (Fig 6).

**Table 1:** Summary of mean water chemistry parameters throughout the study period expressed as mean ± 1 SD.

Target Treatment	Temperature (°C)	pCO_2_ (ppm)	Salinity (ppt)	pH	Ω_a_	TA (μmol kg^-1^ SW)	DIC (μmol kg^-1^ SW)
LT-LCO_2_	26.2 ± 0.16	380 ± 36	32.5 ± 1.3	8.07 ± 0.04	3.6 ± 0.3	2323 ± 71	2012 ± 63
HT-LCO_2_	30.2 ± 0.17	393 ± 52	32.5 ± 1.3	8.06 ± 0.05	4.1 ± 0.4	2357 ± 63	2012 ± 56
LT-HCO_2_	26.1 ± 0.14	789 ± 80	32.5 ± 1.2	7.81 ± 0.04	2.2 ± 0.2	2349 ± 56	2174 ± 54
HT-HCO_2_	30.0 ± 0.19	823 ± 85	32.5 ± 1.3	7.80 ± 0.05	2.5 ± 0.3	2358 ± 61	2164 ± 50

**Table 2:** Results of full-factorial 3-way mixed ANOVA with replicate tank nested within temperature and CO_2_ level on the dependent variables: growth, total lipid content, chlorophyll *a*, and symbiont density.

Factor	Source	Degrees of Freedom	F ratio	p value	Factor	Source	Degrees of Freedom	F ratio	p value
Growth	T	1	7.07	**0.001**	Chlorophyll *a*	T	1	55.1	**<.0001**
	C	1	11.3	**0.001**		C	1	0.83	0.366
	T*C	1	0.37	0.546		T*C	1	0.19	0.662
	F	1	19.9	**<.001**		F	1	8.18	**0.006**
	T*F	1	6.64	**0.012**		T*F	1	0.27	0.603
	C*F	1	3.66	0.060		C*F	1	0.42	0.520
	T*C*F	1	1.43	0.235		T*C*F	1	1.05	0.309
	Tank [T, CO_2_]	4	1.01	0.411		Tank [T, CO_2_]	4	7.49	**<.0001**
Feeding rate	T	1	0.07	0.788	Symbiont density	T	1	40.2	**<.0001**
	C	1	4.44	**0.045**		C	1	0.33	0.571
	T*C	1	0.50	0.485		T*C	1	1.50	0.223
	Tank [T, CO_2_]	4	0.58	0.679					
Total lipid content	T	1	3.05	0.086		F	1	5.05	**0.028**
	C	1	5.63	**0.021**		T*F	1	2.23	0.140
	T*C	1	1.29	0.260		C*F	1	1.34	0.252
	F	1	1.64	0.206		T*C*F	1	0.15	0.701
	T*F	1	0.88	0.351		Tank [T, CO_2_]	4	4.52	**0.003**
	C*F	1	4.14	**0.047**					
	T*C*F	1	1.36	0.249					
	Tank [T, CO_2_]	4	0.92	0.46					

Main effects are temperature (T), carbon dioxide (C) and feeding (F). Results of full-factorial 2-way mixed ANOVA with replicate tank nested within temperature and CO_2_ level is also shown for the dependent variable feeding rate. Significant p values are bolded.

**Table 3:** Analysis of variance for a test of bivariate linear fit between feeding rate and lipid content.

Source	DF	F ratio	P value
Model	1	17.16	**0.0002**
Error	34		
Total	35		

## Discussion

### Growth response is mediated by feeding during stress

In this study we found that the threatened coral, *Acropora cervicornis*, was able to increase its feeding rate and stored energy reserves (total lipid content) when exposed to high CO_2_ conditions at 26°C or 30°C and mitigate reductions in calcification that caused significant decreases in growth rate in unfed corals. To our knowledge, only one previous study has reported the ability of a coral to increase its feeding rate and energy reserves under thermal (but not OA) stress and concurrently exhibit mitigation of depressed calcification. Grottoli et al. [8] found that thermally stressed (bleached) colonies of *Montipora capitata* increased their feeding rate six-fold relative to unstressed controls while two other coral species in the study, *Porites compressa* and *Porites lobata*, did not. Growth rates were not measured as part of that lab study. However, in a related study *M*. *capitata* and *P*. *compressa* corals were bleached in the lab for one month at 30°C and then recovered on the reef where they could feed naturally [34]. Growth rate data was obtained after one and a half, four, and eight months on the reef. After eight months the growth rate of *M*. *capitata* had recovered to the point that it was not significantly different from the unbleached controls, while the growth rate of *P*. *compressa* was still significantly slower than the unbleached controls. The conclusion based on feeding data from [8] and growth data from [34] was that the species that was able to meet its energy needs by switching from an autotrophic to a heterotrophic energy source had a faster recovery from a thermal stress event.

Edmunds [11] performed a factorial experiment similar in design to the present study with two temperatures (26 and 29°C), two CO_2_ levels (416 and 815 ppm), and fed and unfed treatments. He reported that feeding partially ameliorated the effect of high CO_2_ on biomass-normalized growth, but did not measure feeding rates so it is not possible to know if the massive *Porites* species used actively increased its usage of heterotrophy. Previous OA work with *A*. *cervicornis* has shown that at 25°C [35] and 28°C [32], CO_2_ levels between 700–900 ppm elicited decreases in growth rate, but corals in both studies did not have opportunities to feed heterotrophically. It remains unknown what minimum heterotrophy rates are necessary to elicit a stress-buffering response, and therefore corals that do obtain some food, (but perhaps below some hypothetical threshold), may still display depressed calcification. Nonetheless, this work represents the first time a study has shown increases in feeding rate and lipid content, and concurrent mitigation of OA-induced decreases in surface area-normalized calcification. Recent work suggests that food availability may also determine the response of other marine invertebrates to OA. Pansch et al. [46] found that the barnacle *B*. *improvisus* from Sweden was able to withstand elevated CO_2_ conditions (~1,000 μatm) over five weeks when food was plentiful, but showed reduced growth when food was limited. This study provides further evidence that feeding and energy availability can mediate reductions in growth due to OA stress in marine invertebrates.

### Heterotrophy and its relationship to lipids

Feeding rates reported in this study (0.9–1.3 plankters cm^-2^ hr^-1^) are similar to those reported by Palardy et al. [26] (0.70–1.0 plankters cm^-2^ hr^-1^) for *Pocillopora damicornis*, a Pacific species with similar small polyps and branching morphology. Lipid content reported here ranged from 0.8–1.4 mg lipid cm^-2^ similar to what Anthony et al. [4] reported for *Acropora intermedia* (0.5–2.0 mg lipid cm^-2^). The present data show a significant effect of CO_2_ and significant interaction between CO_2_ and feeding on lipid content with the highest mean lipids in fed corals at elevated CO_2_. This increase is consistent with Schoepf et al. [36] who showed that lipid content in *Acropora millepora* also increased with elevated CO_2,_ but not temperature. The current results differ, however, from those of Drenkard et al. (2013) who also tested the impact of feeding and CO_2_ on larvae of the Atlantic coral *F*. *fragum*. While Drenkard et al. did not directly measure feeding rate, they found that lipid content was not significantly affected by CO_2_ or feeding in juvenile corals [45]. These contrasting lipid responses highlight that juvenile and adult corals may respond differently under OA conditions. For example, juveniles may preferentially invest in tissue growth, while adults may invest in lipid storage [45], as the present data suggest. Heterotrophy and lipid content appear to be linked in *A*. *cervicornis*, as both metrics increased in response to elevated CO_2_, and there was a significant correlation between the two variables (Fig 6). This correlation is consistent with Teece et al. [6], who state that heterotrophy can be a direct source of certain types of lipids for the coral host. These concurrent increases provide evidence that elevated CO_2_ is a metabolic stress to *A*. *cervicornis*, necessitating the host to increase feeding rate and subsequently lipids.

Increased *A*. *cervicornis* heterotrophy at 800 ppm, but not at 30°C, suggests that the elevated CO_2_ level used was potentially stressful enough to elicit a feeding response, whereas the severity and/or duration of the elevated temperature level may not have been enough to necessitate a feeding response. Perhaps in order for temperature stress to induce a feeding/lipid response, the coral must already be at or above the bleaching threshold for a certain amount of time. We do show, however, that being fed versus unfed ameliorated the potentially stressful effect of elevated temperature on growth (Fig 1) even though feeding rate did not increase significantly at elevated temperature. The hypothesis that these corals were close to their thermal stress threshold is supported by the observations that chlorophyll *a* content and symbiont density were both reduced in the 30°C treatments (Figs 4 and 5), indicating that the corals were likely experiencing the early stages of bleaching and were likely deriving less energy from their symbionts, although seemingly not dramatically enough to necessitate a significant increase in feeding rates.

### Heterotrophic plasticity in corals—driven by metabolic stress thresholds?

Based on the reliance a coral has on its symbionts for daily metabolic energy, one might have predicted an increase in heterotrophy as a result of reductions in symbiont density and chlorophyll *a* level in order to compensate for potential losses in photosynthate transferred to the host. We show, however, that responses are more nuanced than this. Significant decreases in symbiont density and chlorophyll *a* at high temperature were observed without seeing an increase in feeding rate, and conversely no significant decreases in symbiont density and chlorophyll *a* at high CO_2_ were accompanied by increases in feeding rate. These results suggest that in *A*. *cervicornis*, increases in feeding rate are not related to or driven by symbiont density and chlorophyll *a* content. Instead, we propose that increased feeding is driven by exposure to the metabolic stress caused by elevated CO_2_ which increases the energetic expense incurred by the Ca^+2^-H^+1^-ATPase pump at the site of calcification [37,38,39,48]. Although the energetic cost of calcification is not known accurately, it has been estimated to be on the order of 30% of the total energy budget [40,41]. Taking the view that coral calcification is an energy-demanding process and that the saturation state of the calcifying fluid starts out close to that of seawater [42,43], it stands to reason that corals will have to expend more energy to achieve a constant rate of calcification as the saturation state of the ambient seawater decreases due to OA. Following this line of logic, one might expect that energy-replete corals might be able to buffer decreases in saturation state of the ambient seawater over a certain range by devoting more energy to proton pumping. If a coral is energetically compromised due to loss of symbionts, lack of success at heterotrophy, or depleted lipid reserves, then it may be forced to devote less energy to proton pumping with the consequence that calcification declines. Our data support the idea that without supplemental energy for this mechanism under stress, growth will suffer; conversely, when provided with additional energy from heterotrophy, growth will not decrease significantly from ambient rates.

In addition to feeding potentially being driven by level of coral energy reserves, the use of heterotrophy to compensate for a stressor may also be species-specific. Grottoli et al. [8] found that 30°C was sufficient to cause increased feeding in the Hawaiian coral *Montipora capitata* recovering from bleaching. Ferrier-Pages et al. [44] found that feeding rates of three species of corals varied in their responses to the same elevated temperature stress of 31°C. These studies and the present study highlight the validity of accounting for heterotrophy when assessing coral physiological response to climate change stress. More research is needed to better understand exactly what drives increased feeding in the host. Kaniewska et al. [13] proposed a model of cell-wide responses of corals to OA in which they describe how elevated CO_2_ causes disruption to host mitochondria, decreasing cellular metabolism and increasing lipid oxidation, which could necessitate feeding to replenish cellular lipids. It is difficult to determine what rate of heterotrophy can replenish lipids to a degree whereby corals display resilience to stress. To determine this, one would have to assess the severity and duration of the stress event, the amount of lipid reserves in the coral prior to the stress event, and the degree of heterotrophy the coral is capable of [9]. Further research is necessary to determine if increased heterotrophy and lipid content under elevated CO_2_ can mitigate decreases in calcification under longer-term scenarios greater than eight weeks.

This study provides a glimmer of hope that a critically endangered species with access to food sources other than photosynthate may be able to maintain growth physiology under climate change stress. Resilience in the wild will be dependent on reefs with naturally high zooplankton abundance. Therefore, decisions concerning the placement of MPAs and/or coral nurseries may benefit from the careful consideration of natural food availability at the proposed sites. Moreover, future physiological studies on coral responses to climate change should consider assessing heterotrophy and lipid content.
