# Correction: Threatened Caribbean Coral Is Able to Mitigate the Adverse Effects of Ocean Acidification on Calcification by Increasing Feeding Rate

**DOI:** 10.1371/journal.pone.0139398

**Published:** 2015-09-24

**Authors:** Erica K. Towle, Ian C. Enochs, Chris Langdon

There are errors in the eleventh and twelfth sentences of the Feeding Rate subsection of the Materials and Methods section. The correct sentence should be: This concentration was chosen to mimic a level close to that used in Edmunds [11], ca. 1.6 x 104 nauplii L-1, in order to provide corals with ad libitum access to zooplankton in order to see any potential differences in feeding due to thermal and CO2 stress.
